# Bibliometric and visualized analysis of the application of nanotechnology in glioma

**DOI:** 10.3389/fphar.2022.995512

**Published:** 2022-09-15

**Authors:** Xue Du, Chunbao Chen, Lu Yang, Yu Cui, Bangxian Tan

**Affiliations:** ^1^ Department of Oncology, Afliated Hospital of North Sichuan Medical College, Nanchong, China; ^2^ North Sichuan Medical College, Nanchong, China

**Keywords:** bibliometric, glioma, blood–brain barrier, nanotechnology, nanoparticles, drug delivery

## Abstract

**Background:** Glioma is the most prevalent malignant tumor in the central nervous system (CNS). Due to its highly invasive characteristics and the existence of the blood–brain barrier (BBB), the early diagnosis and treatment of glioma remains a major challenge in cancer. With the flourishing development of nanotechnology, targeted nano-therapy for glioma has become a hot topic of current research by using the characteristics of nanoparticles (NPs), such as it is easier to pass the blood–brain barrier, degradable, and aids controllable release of drugs in the brain. The purpose of this study is to visualize the scientific achievements and research trends of the application of nanotechnology in glioma.

**Methods:** We searched the literature related to glioma nanotechnology on the Web of Science (WOS). The bibliometric and visual analysis was performed mainly using CiteSpace, VOSviewer, and R software, for countries/regions, authors, journals, references, and keywords associated with the field.

**Results**: A total of 3,290 publications from 2012 to June 2022 were searched, and 2,041 works of literature were finally obtained according to the search criteria, the number of publications increasing year by year, with an average growth rate (AGR) of 15.22% from 2012 to 2021. China published 694 (20.99%), followed by the United States (480, 20.70%). The institution with the highest number of publications is Fudan Univ (111, 13.16%), and 80% of the top ten institutions belong to China. HUILE GAO (30) and XINGUO JIANG (30) both published the largest number of research studies. STUPP R (412) was the most cited author, followed by GAO HL (224). The degree of collaboration (DC) among countries/regions, research institutions, and authors is 23.37%, 86.23%, and 99.22%, respectively. International Journal of Nanomedicine published the largest number of publications (81), followed by Biomaterials (73). Biomaterials (1,420) was the most cited journal, followed by J Control Release (1,300). The high frequency of keywords was drug delivery (487), followed by nanoparticle (450), which indicates that nanoparticles (NPs) as a carrier for drug delivery is a hot topic of current research and a direction of continuous development.

**Conclusion:** In recent years, nanotechnology has attracted much attention in the medical field. Cooperation and communication between countries/regions and institutions need to be strengthened in future research to promote the development of nanomedicine. Nanotherapeutic drug delivery systems (NDDS) can enhance drug penetration and retention in tumor tissues, improve drug targeting, and reduce the toxic side effects of drugs, which has great potential for the treatment of glioma and has become the focus of current research and future research trends in the treatment of glioma.

## Introduction

Glioma is the most common primary malignant tumor of the CNS, accounting for 30% of all primary brain tumors and 80% of malignant tumors, and is the main cause of death in primary brain tumors (Chen et al., 2017). Due to the rapid proliferation and high invasiveness of gliomas, which are resistant to current therapies (Xun et al., 2021), the traditional treatment of glioma includes maximum surgical resection, radiotherapy, and temozolomide (TMZ) chemotherapy. However, the overall prognosis of malignant gliomas is poor due to incomplete surgical resection, the presence of an immunosuppressive tumor microenvironment (TME), and the blood–brain barrier (BBB) that hinders effective drug treatment (Zarnett et al., 2015). The BBB is an important transport barrier that controls the exchange of substances between the blood and the CNS and maintains homeostasis within the CNS (Scott et al., 2002). The BBB can prevent harmful substances from entering the brain tissue from the blood, protect the brain from running in a stable environment, and prevent most drugs from entering the brain tissue from the blood, limiting the diagnosis and treatment of intracranial diseases (Carmeliet and De Strooper, 2012; Lipsman et al., 2018). Therefore, how to effectively make drugs cross the BBB has become a major challenge in clinical diagnosis and treatment of brain diseases (Dong et al., 2022).

With the introduction of the term “Nanoneurosurgery”, neurosurgery has entered a completely new era (Dunn and Black, 2003), neurosurgeons can intervene at the molecular level, and such molecular therapies can complement existing conventional treatments, providing a new strategy for the diagnosis and treatment of malignant gliomas (Ljubimov et al., 2022). In recent years, nanotechnology has developed rapidly, and there have been huge breakthroughs in the field of medical biology (Jiang and Pu, 2018). A variety of inorganic/organic/natural nanomaterials with BBB-targeting ligands and/or cell-penetrating peptides (CPPs) surface modifications have been developed to span the BBB for high-precision brain tumor therapy (Tang et al., 2019); this nanotechnology across the BBB is expected to revolutionize the traditional treatment of gliomas. Magnetic resonance imaging (MRI), computed tomography (CT), and positron emission tomography (PET) are the most commonly used methods for the diagnosis and characterization of brain tumors. However, these conventional examination methods also have many shortcomings, and researchers are constantly developing new imaging techniques to obtain better sensitivity and specificity, higher temporal and spatial resolution and deep tissue penetration (Kircher et al., 2012; Gao et al., 2017). Recently, well-designed nanoprobes have provided great opportunities for the development of other imaging modalities (Tang et al., 2019). In addition, what is even more exciting is the emerging nanotechnology across the BBB, which integrates the dual functions of therapy and imaging (Kircher et al., 2012; Bao et al., 2018; Zhou et al., 2018).

At present, there is no research work on using bibliometric methods to analyze the application of nanotechnology in gliomas. A bibliometric analysis of the application of nanotechnology in glioma may provide more insight into the role of nanotechnology in the diagnosis and treatment of glioma. CiteSpace is a tool for visual analysis of academic literature in a research field (Chen, 2006). Based on statistical and quantitative analysis, researchers can obtain useful information on future research directions and trends (Wei et al., 2020). The purpose of this study is to comprehensively analyze the research and application of nanotechnology in glioma from multiple perspectives by using bibliometrics tools and comprehensively analyze the development status and future research trends and hotspots in this field.

## Materials and methods

### Data source

**FIGURE 1:**
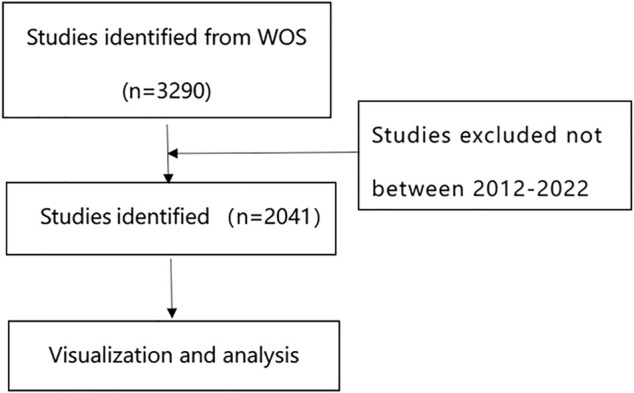
Flowchart of literature selection.

In June 2022, we searched the WOS online database, and the time span of the search dates was from 2012 to June 2022. The search strategy was as follows: (TS=(glioma*) OR TS=(glioblastoma*) OR TS=(astrocytoma) OR TS = (malignant glioma) OR TS = (glioblastoma multiform*) OR TS=(gliosarcoma)) AND (TS=(nanotechnology) OR TS = (nanomaterials) OR TS=(nanoparticles)). The inclusion criteria for the literature were as follows: 1) research on nanotechnology in glioma was the theme; 2) the type of literature included articles and reviews and freely available data; 3) the language of the literature was English. The exclusion criteria were as follows: 1) the articles were not related to the research theme; 2) the articles were conference abstracts, news, or briefs. To ensure the quality of the search, the complete literature was evaluated by two reviewers, and any disagreements were resolved through discussion until consensus was reached. Figure 1 shows the flow chart of the literature selection process.

### Analysis Method

We mainly used CiteSpace, VOSviewer, and R software for visual analysis and Microsoft Excel 2019 for data management. CiteSpace software is a document visualization tool developed by Prof. Chaomei Chen to analyze indicators such as country/region, author, institution, journal, and reference (Chen and Song, 2019). CiteSpace is also used to analyze outbreak keywords to predict trends in the field of research (Ma et al., 2022). VOSviewer is a software for building and viewing bibliometric maps. The main purpose of developing VOSviewer is to analyze bibliometric networks and construct visual network maps, ultimately achieving an in-depth and comprehensive understanding of the structure and dynamic development of scientific research (van Eck and Waltman, 2010). In a visual map, the node size represents the frequency of occurrence, with larger nodes representing a higher frequency of occurrence. The connections between nodes represent collaboration or co-occurrence relationships. Betweenness centrality is an important parameter in Citespace; in general, centrality ≥0.1 is considered a more important node, and Citespace also marks it with a purple circle. It mainly measures the value of the node acting as a bridge in the whole network structure (Da et al., 2021).

### Statistical analysis

SPSS (IBM SPSS Statistics 27) was utilized for statistical processing of data. *p* < 0.05 hinted a statistically significant difference.

## Results

### Publishing trend

**FIGURE 2:**
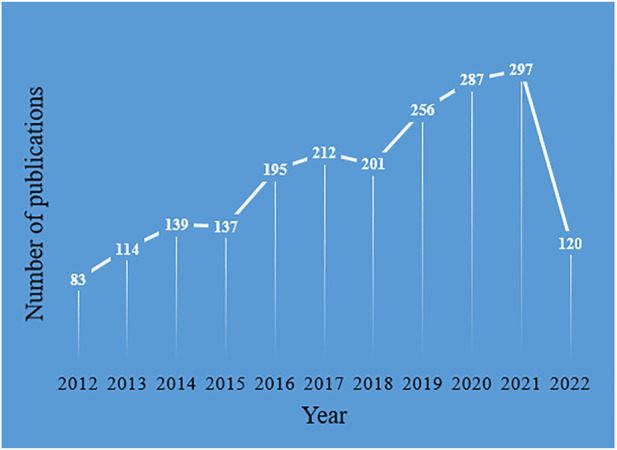
Number of annual publications.

From 2012 to June 2022, WOS searched a total of 3,290 publications about nanotechnology research in gliomas, and 2,041 were finally obtained according to the search criteria. Among them, 1,745 were articles (85.50%), and 296 were reviews (14.50%). Figure 2 shows the trend of the number of publications per year for the period 2012 to June 2022. The publication volume began to increase from 2012 to 2015, increased steadily from 2016 to 2018, exploded from 2019 to 2021, and reached 297 in 2021, with an average growth rate (AGR) of 15.22%. The compound annual growth rate (CAGR) of publications gradually decreased from 50.06% in 2013 to 36.91% in 2021 (Santha Kumar and Kaliyaperumal, 2015), as seen both in Supplementary Table S1 and Supplementary Figure S1. This indicates that though the yearly output is increasing year after year, the CAGR is in a downward trend. As seen in Supplementary Table S2 and Supplementary Figure S2, the relative growth rate (RGR) decreased from 2013 (86%) to 2021 (17%). There exists a direct equivalence between the relative growth rate and the doubling time (Santha Kumar and Kaliyaperumal, 2015). The doubling time (DT) increases when calculated on an annual basis. From Supplementary Table S2 and Supplementary Figure S3, it can be seen that the DT increased from 0.80 in 2013 to 4.13 in 2021. In addition, we performed Pearson correlation analysis to test the correlation between publications and citations by Pearson correlation coefficient, and a *p*-value < 0.05 was considered a significant correlation. Our analysis showed results that there was a high positive correlation between publications and citations (r = 0.854, *p* < 0.001).

### Distribution of countries/Regions and institutions

A total of 2,041 publications from 79 different countries/regions and 2,247 institutions were published from 2012 to June 2022. The highest number of publications was from China (694, 20.99%), followed by the United States (480, 20.70%), much higher than other countries (Table 1). The research institution with the most publications was Fudan Univ (111, 13.16%), and 80% of the top ten institutions belonged to China (Table 1). Among these countries/regions, the United States (0.49), France (0.2), the People’s Republic of China (0.19), Italy (0.13), Spain (0.11), and Germany (0.11) showed a high centrality. In addition, we analyzed the degree of collaboration (DC) between countries/regions and research institutions, with DC of 23.37 and 86.32%, respectively.

**TABLE 1:** Top ten countries/regions and institutions for related publications.

Rank	Count	Centrality	Countries/regions	Rank	Count	Centrality	Institutions
1	694	0.19	People’s Republic of China	1	111	0.2	Fudan Univ
2	480	0.49	United States	2	61	0.14	Chinese Acad Sci
3	137	0.07	India	3	54	0.06	Shanghai Jiao Tong Univ
4	111	0.2	France	4	37	0.04	Sichuan Univ
5	103	0.13	Italy	5	32	0.06	Nanjing Med Univ
6	88	0.09	Iran	6	31	0.04	Johns Hopkins Univ
7	78	0.05	South Korea	7	29	0.04	Soochow Univ
8	70	0.11	Spain	8	29	0.03	Southeast Univ
9	69	0.01	Taiwan	9	27	0.1	Russian Acad Sci
10	67	0.11	Germany	10	21	0.1	Sun Yat Sen Univ

**FIGURE 3:**
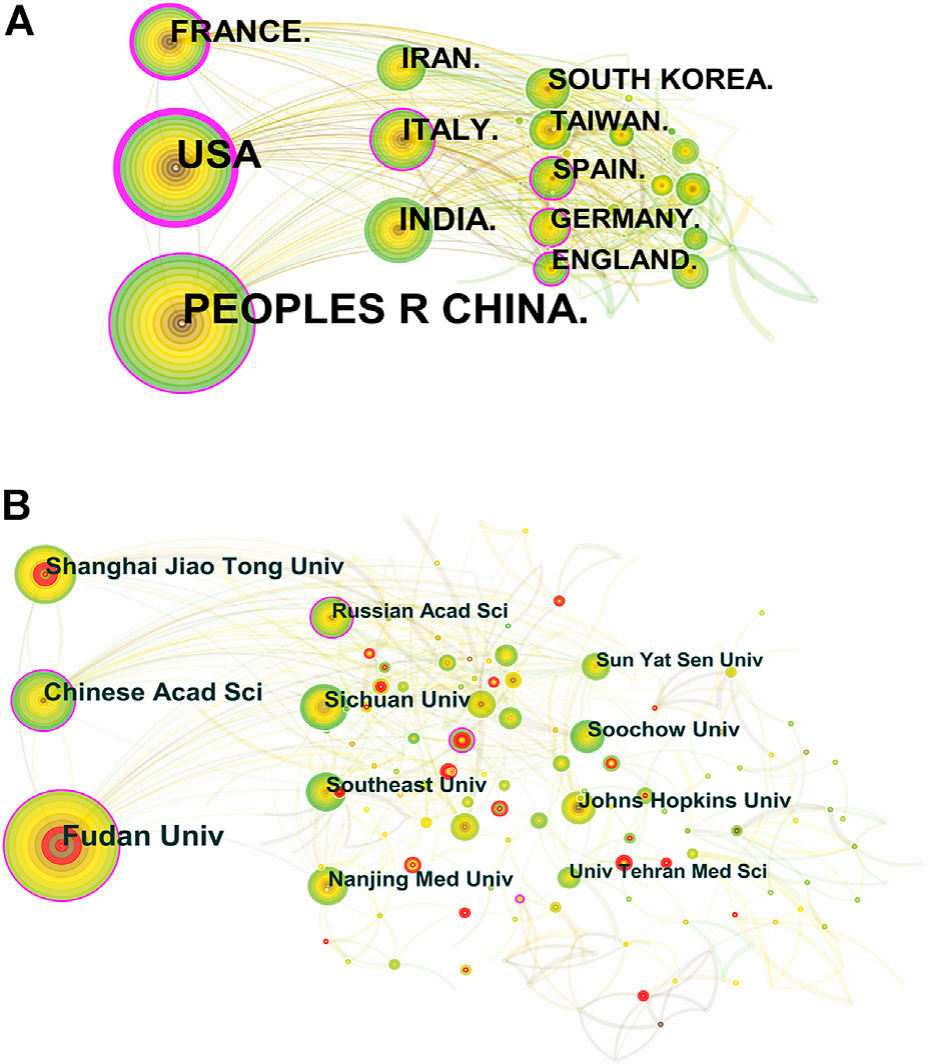
**(A)** Distribution of publications on nanotechnology in glioma research from different countries/regions. **(B)** Distribution of publications on nanotechnology in glioma research from different institutions.

In the CiteSpace visualization mapping, each circle represents a country/region or institution, the size of the circle indicates the publication output of this country/region or institution, the line between the circles indicates the collaboration between countries/regions or institutions, the nodes with high centrality are shown as purple rings, and the thickness of the purple ring describes the size of the value of mediated centrality (Figures 3A and B).

### Authors and Co-Cited Authors

**FIGURE 4:**
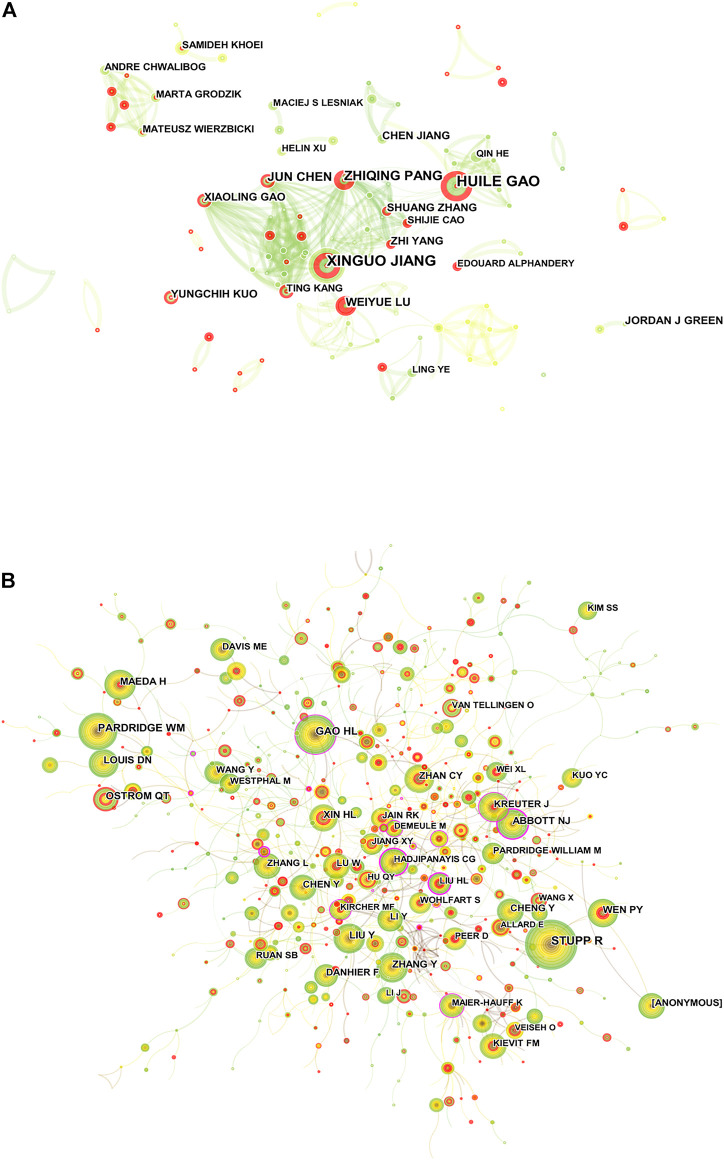
**(A)** Visualization map of Citespace between authors of related publications. **(B)** CiteSpace visualization map of co-cited authors.

A total of 9,714 researchers participated in the publication of related literature, and the productivity per author from 2012 to June 2022 was 0.21 (Santha Kumar and Kaliyaperumal, 2015). HUILE GAO (30) and XINGUO JIANG (30) published the most research studies, followed by ZHIQING PANG (19) and JUN CHEN (17) (Supplementary Table S3). Figure 4A shows the visual analysis mapping of the author cooperation network; each circle represents an author, the larger the circle indicates more postings, the line between represents the connection between authors, and the thicker the connecting line indicates a closer cooperation relationship. The degree of collaboration (DC) between authors was 99.22%. Co-cited authors means that two or more authors are cited by one or more articles at the same time, and these two or more authors constitute a co-citation relationship (Zhang et al., 2021). Among the cited authors listed in VOSviewer, the number of authors with at least one citation is 46,066. STUPP R (412) was the most cited author, followed by GAO HL (224), and ABBOTT NJ (0.27) had the highest centrality, followed by GAO HL (0.16) (Supplementary Table S3). Figure 4B shows a visual network map of the relationship between co-cited authors.

### Journals and co-cited journals

**FIGURE 5:**
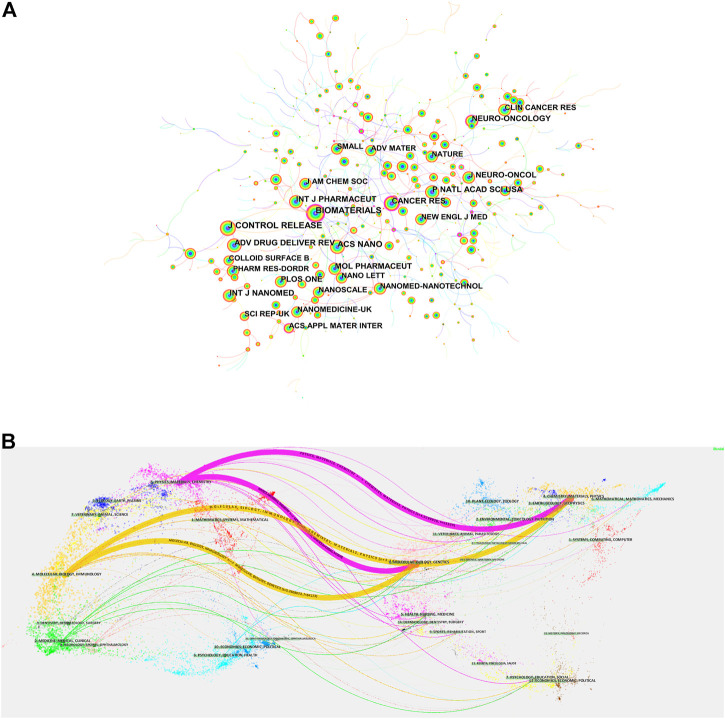
**(A)** Visual mapping of co-cited journals of related literature. **(B)** Dual-map overlay of related publications journals.

We used the “bibliometrix” package in R software (version 4.1.3) to visually analyze the journals of the publication. International Journal of Nanomedicine was the journal with the highest number of published articles (81), followed by Biomaterials (73), and among the top ten academic journals, the highest impact factor (IF) was ACS Nano, with an IF of 18.027 (Table 2). The most cited of the 792 co-cited journals was Biomaterials (1,420), followed by J Control Release (1,300) (Table 3). Among them, Biomaterials has the highest citation frequency and centrality, indicating that this journal has a high core position in this research field. Figure 5A shows a visual map of co-cited journals, with the size of the circles representing the frequency of co-citations and the purple circle indicating higher centrality.

**TABLE 2:** Top ten journals publishing related publications.

Rank	Count	Journals	If 2022	JCR
1	81	International Journal of Nanomedicine	7.033	Q2
2	73	Biomaterials	15.304	Q1
3	64	International Journal of Pharmaceutics	6.51	Q1
4	60	Acs Appplied Materials and Interfaces	10.383	Q1
5	56	Journal of Controlled Release	11.467	Q1
6	46	Nanoscale	8.307	Q1
7	38	International Journal of Molecular Sciences	6.208	Q2
8	37	Nanomedicine	6.096	Q2
9	35	Rsc Advances	4.036	Q2
10	34	Acs Nano	18.027	Q1

**TABLE 3:** Top ten co-cited journals for related publications.

Rank	Count	Centrality	Co-cited journals	IF2022	JCR
1	1,420	0.3	Biomaterials	15.304	Q1
2	1,300	0.1	J Control release	11.467	Q1
3	1,034	0.03	Acs Nano	18.027	Q1
4	958	0.03	Adv Drug Deliver Rev	17.873	Q1
5	936	0.23	Cancer Res	13.312	Q1
6	895	0.03	Int J Nanomed	7.033	Q1
7	875	0.04	P Natl Acad Sci United States	12.779	Q1
8	856	0.07	Int J Pharmaceut	6.51	Q1
9	736	0	Plos One	3.752	Q2
10	732	0	Mol Pharmaceut	5.364	Q1

The dual-map overlay of journals is a way to display information about the distribution, citation trajectory, and drift of the center of gravity of research studies across disciplines (Chen and Leydesdorff, 2014). On the left is the journal distribution where the citing literature is located, and on the right is the journal distribution corresponding to the cited literature (Chen, 2017). The colored paths in Figure 5B indicate the cited relationships, and the yellow paths indicate that literature published in molecular/biology/immunology journals is frequently cited by molecular/biology/genetics journals.

### Co-cited references and references burst

Co-citation is a research method that measures the degree of relevance between research studies and is defined as two or more articles that are cited by one or more research studies at the same time, and these two articles are regarded as co-citation relationship (Ma et al., 2021). Among the 190 co-cited references retrieved, we listed the top ten co-cited references (Supplementary Table S4). Among them, Louis et al. (2016) published “The 2016 World Health Organization Classification of Tumors of the Central Nervous System: a summary”, which was the most frequently cited (70), for the first time, molecular pathological targets were added to the traditional pathological light microscopy morphological diagnosis to achieve precise tumor classification, which helps clinical judgment of prognosis and treatment options. Second, van Tellingen et al. 92015) published “Overcoming the blood–brain tumor barrier (BBTB) for effective glioblastoma treatment”; it reviewed the basic principles of the BBTB, which helps to design tumor-appropriate therapies. In addition, according to the titles of the top ten co-cited literature, it can be understood that their subjects were mainly about the research on the treatment of brain tumors by overcoming the BBTB and BBB through NPs and nanocarriers.

Citation burst analysis can help researchers to identify literature that has received focused attention in a certain time period (Lu et al., 2020). According to the reference strongest citation burst, Supplementary Figure S4 shows that the first citation burst started in 2011 and has been consistently increasing in the last ten years. The top 25 references had citation strengths from 9.7 to 17.73, with the strongest citation strength being Xin et al. (2012) published in “Biomaterials”, a study on anti-glioblastoma efficacy and safety of paclitaxel-loading Angiopep-conjugated dual targeting PEG-PCL nanoparticles.

### Keyword visualization analysis

Keywords are the core of a research article, and the analysis of keywords can summarize the research themes in a particular field and explore hot spots and research directions (Ma et al., 2021). The keywords that appear with high frequency in studies on nanotechnology in glioma are displayed in Supplementary Table S5, including drug delivery (487), nanoparticle (450), *in vitro* (339), delivery (285), cancer (285), blood–brain barrier (266), and indicates the current research hotspots in this field.

**FIGURE 6:**
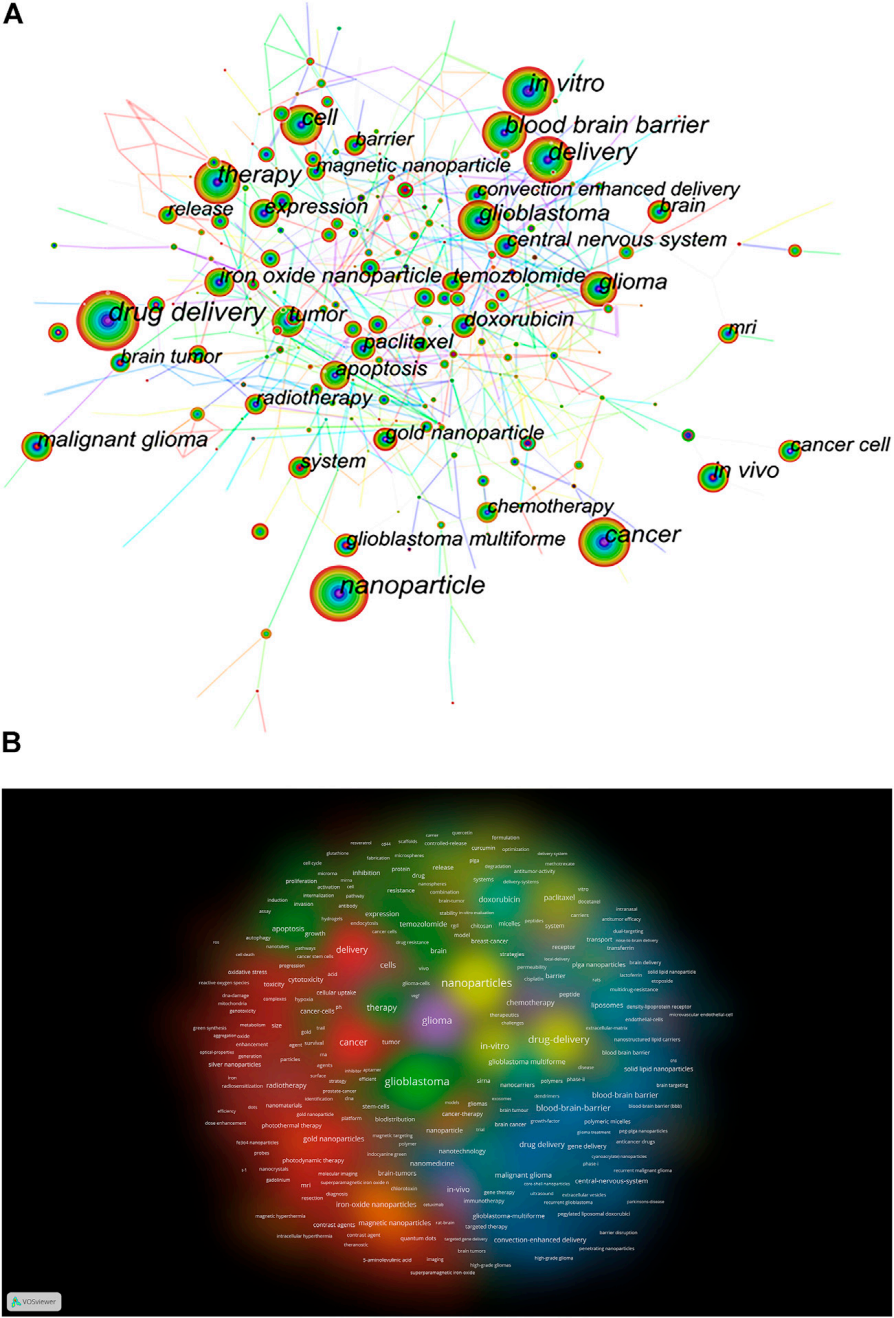
**(A)** Keyword co-occurrence visualization map. **(B)** VOSviewer visualization for keywords density plot.

There are 460 nodes with 2,211 links in the keyword co-occurrence visualization map (Figure 6A). Each node corresponds to a keyword, and the larger the node is, the more frequently the keyword appears; the number of links between nodes and the distance between nodes reflect the tightness of the keywords. The keyword burst visualization map showed the burst intensity of the top 25 keywords (Supplementary Figure S5), the blue line indicated the timeline, and the red part of the blue timeline indicated the time interval of the burst.

Based on the analysis of keyword co-occurrence, the cluster analysis of the network map can reflect the basic knowledge structure of related research fields. We used VOSviewer software to perform co-occurrence clustering analysis and density visualization of keywords in the literature. Supplementary Figure S6 shows that there are eight clusters of red, green, blue, yellow, purple, cyan, orange, and brown, representing eight different research directions. The major keywords for red clusters are gold NPs, delivery, cancer, and toxicity. The keywords in the green cluster are glioblastoma, temozolomide, expression, and apoptosis. Keywords for the blue cluster include blood–brain barrier, malignant glioma, drug delivery, and solid–lipid NPs. Keywords for the yellow cluster include NPs, plag NPs, paclitaxel, and efficacy. keywords in the purple cluster mainly include chemotherapy, *in vivo*, and trial. Keywords for cyan cluster mainly include doxorubicin, liposomes, barrier, and transport. Keywords of the orange cluster include iron-oxide NPs, carbon nanotubes, and hyperthermia. Keywords for brown clusters mainly include cells and radiation therapy. Figure 6B shows the keywords clustering density plot by VOSviewer.

## Discussion

### General information

The number of publications is one of the important indicators to measure the hotness and development speed of academic research in a certain period of time, which is important for analyzing the research dynamics and predicting the development trend. Figure 2A shows that the total number of publications is on the rise. The AGR of publications was 15.22%. Although the number of publications increased year by year, CAGR and RGR decreased, and DT increased. This indicates that research on nanotechnology in glioma is developing. In 2012, Ren et al. (2012) successfully constructed a dual-targeting drug delivery system based on PEGylated oxidized multi-walled carbon nanotubes (O-MWNTs) modified with angiopep-2 (O-MWNTs-PEG-ANG). It also proved that O-MWNTs-PEG-ANG has good biocompatibility and low toxicity and is a promising dual-targeting vector for the treatment of brain tumors. Nanocarriers, as emerging carriers in drug delivery systems, are able to change the drug transport capacity across the BBB and improve the targeting efficiency of glioma treatment. Furthermore, with the development of nanotechnology, the burgeoning cancer nanotechnology is expected to use multifunctional NPs for imaging diagnosis and targeted therapy of glioma, providing new strategies for the diagnosis and treatment of gliomas (Nduom et al., 2012). Thus, nanotechnology in glioma has received increasing attention from researchers and has gradually become a research hotspot in the field of glioma.

The ranking of the number of research articles issued by countries/regions and institutions can objectively reflect the research level and influence of the relevant countries/regions and countries in the relevant research fields. The results of this study showed that the highest number of research publications related to nanotechnology in glioma from 2012 to 2022 was from China (694), followed by the United States (480), indicating that China and the United States are the major scientific exporters in this research field and have contributed significantly to the development of the field. Centrality is a measure of the importance of nodes in a network and is mainly used to measure the value of the bridging function of nodes in the whole network structure (Da et al., 2021). Among the top ten countries/regions, the United States has the highest centrality (0.49), which means it plays a key bridging role in the global network of countries/regions cooperation. The DC between countries/regions is 23.37%. The main publishing institutions are universities from various countries, and the top five institutions are all from China, namely, Fudan Univ, Chinese Acad Sci, Shanghai Jiao Tong Univ, Sichuan Univ, and Nanjing Med Univ. The DC between research institutions was 86.23%. Strengthening academic exchange and communication between countries/regions and research institutions closely in future research will contribute to the development of this research field.

Among the 9,714 researchers, HUILE GAO (30) and XINGUO JIANG (30) have the highest number of publications, which demonstrates their influence in the field. HUILE GAO et al. (Gao et al., 2012) used phage-displayed TGN peptides and AS1411 aptamers as specific targeting ligands for BBB and cancer cells, respectively, and combined them with NPs to establish a brain glioma cascade delivery system (AsTNP). *In vitro* cellular uptake and three-dimensional (3D) tumor sphere penetration studies have shown that the system can not only target vascular endothelial cells and tumor cells but also penetrate the endothelial monolayer and tumor cells to reach the core of the tumor sphere. It is obvious that nanomedicines demonstrate a bright prospect in the treatment of glioma. Among the co-cited authors, STUPP R (412) had the highest number of citations, followed by GAO HL N (224).

The impact factor (IF) of journals is widely accepted and recognized internationally and has become an important indicator for evaluating the academic influence of journals. International Journal of Nanomedicine published the largest number of research studies (81), followed by Biomaterials (73). It can be seen that the study of nano-medical biomaterials is not only the current research focus but also the development trend in the future. In terms of co-cited journals, Biomaterials (1,420) was the most cited journal, followed by J Control Release (1,300) and ACS Nano (1,034).

Reference co-citation analysis can find the important articles that form each research clustering theme. The top ten co-cited research studies of nanotechnology in glioma-related studies were mainly focused on NPs and BBB. This suggests that overcoming the BBB through NPs for the treatment of intracerebral diseases is a subject of intense research in this field. In the reference burst analysis, Xin et al. (2012) proposed Angiopep-conjugated PEG-PCL nanoparticles (ANG-PEG-NP) as a dual targeting drug delivery system for glioma treatment, which showed the strongest bursts of the highest strength. It can be seen that nano-targeted drug delivery systems show broad application prospects in the treatment of glioma.

### Research hotspots

Keywords are used to express the subject information of the article, and they are the refinement and essence of the central content of the article. the analysis of the frequency of keyword occurrence can directly reflect the research hotspots and development trends in a certain discipline field. The keywords that appeared with high frequency in this research field, shown in Table 8, included drug delivery (487), nanoparticle (450), *in vitro* (339), delivery (285), cancer (285), blood–brain barrier (266). Cluster analysis was performed based on keyword co-occurrence, resulting in eight color clusters representing different research parties. The research hotspots and trends of nanotechnology in glioma were identified. The main contents are as follows:

(1) Nanoparticles for drug delivery

Therapeutic approaches using nanomedicine have been proven to facilitate drug crossing the BBB and maintain drug biological distribution and accumulation at the target location (Neganova et al., 2022). Targeted drug delivery system (TDDS) is a system that can release drugs in a controlled manner from a pre-selected biological site. The advantages of nanoparticle-based nanotherapeutic drug delivery systems (NDDS) include extended half-life, improved biodistribution, increased drug circulation time, and controlled and sustained drug release (Jain et al., 2015). Based on these advantages, more and more researchers are focusing on the construction of nanocarriers for drug delivery to overcome biological barriers as well as anti-drug and drug resistance. Currently developed nanoparticle drug-carrying particles include liposomes NPs (Sercombe et al., 2015), polymers NPs (Afsharzadeh et al., 2018), and inorganic NPs (Yang et al., 2019). Liposome NPs are widely used in the delivery of nucleic acids (Leung et al., 2015), with simple synthesis, small size, and serum stability. However, despite these advantages, the liposome NPs system is still limited by low drug loading and biological distribution, resulting in high uptake rates in the liver and spleen (Fenton et al., 2018). Polymeric NPs are ideal drug carriers for co-delivery applications (Afsharzadeh et al., 2018), with biodegradability, water solubility, biocompatibility, biomimetic properties, and storage stability. However, the disadvantages of polymeric NPs include an increased risk of particle aggregation and toxicity. Inorganic materials such as gold, iron, and silica have been used to synthesize nanostructured materials with a wide variety of sizes, structures, and geometries (Singh et al., 2018) and with unique physical, electrical, magnetic, and optical properties (Arias et al., 2018; Wang et al., 2019). Due to the magnetic, radioactive, and plasmonic properties of inorganic NPs, they offer unique advantages in applications such as diagnostics, imaging, and photothermal therapy. However, their clinical application is limited due to low solubility and toxicity (Manshian et al., 2017; Arias et al., 2018). Although some nano-drugs were approved and used in the clinic, there are still problems such as biological distribution, delivery efficiency, and toxicity risk. Improving biological distribution, enhancing aggregation and delivery efficiency at target sites, improving safety, and reducing toxicity are the focus and hot spots of future research on nano-targeted drug delivery.

(2) Nanotechnology for imaging and diagnosis in glioma

Highly infiltrative and aggressive glioma cells obscure the border between the tumor and normal brain tissue, making precise diagnosis and complete resection extremely difficult. Nanomaterials have good penetration and tumor enrichment effect and can be used for real-time, dynamic, and visual tumor radiography. As a molecular probe, it can be used in many imaging modes, such as magnetic resonance, nuclear medicine, optical imaging, and the integration of diagnosis and treatment. Wang et al. (2020) reported a new two-way magnetic resonance tuning (t-MRET) nanoprobe for quantitative imaging of molecular targets in tumors and sensitive detection of very small intracranial tumors in patient-derived xenograft models. Yang et al. (2020) reported a biomimetic catalase-integrated-albumin phototheranostic nanoprobe (ICG/AuNR@BCNP) to realize brain multimodal imaging, amplified phototherapy, and guided operation of gliomas. Jia et al. (2019) constructed indocyanine green (ICG) biomimetic proteolipid NPs and showed that such biomimetic proteolipid NPs are promising phototheranostic nanoplatforms for brain tumor–specific imaging and therapy. Reichel et al. (2020) reported a near-infrared fluorescence (NIRF)–based tumor boundary visualization and image-guided drug delivery into GBM tumors with a fluorescent nanoparticle platform. It is evident that, in recent years, the development and application of nanotechnology in biomedicine, especially in tumor imaging and diagnosis, and guided surgery have received wide attention. Therefore, the development of nanomaterials for the integration of tumor multimodal imaging and diagnosis and treatment is not only the research trend and hot spot of glioma in the future but also the new direction of the development of tumor nanomedicine in the future.

(3) Nanomedicine for the treatment of glioma

Drug delivery to the brain is greatly hindered by the presence of the BBB, but breakthroughs in nanotechnology have yielded multifunctional theranostic nanoplatforms that can cross or bypass the BBB, making effective treatment of gliomas possible (Tang et al., 2019). To reach the CNS, NPs must be absorbed by endothelial cells of the BBB by receptor-mediated endocytosis and then excreted to the other side (von Roemeling et al., 2017; Saraiva et al., 2016). Receptor-mediated cell transfer is an effective way to deliver therapeutic drugs into the brain or infiltrate tumor tissue (Zhou et al., 2019). Among the molecules being developed to deliver NPs, transferrin receptors are theoretically more advantageous than other transporter types, but no clinical success has been seen yet (Johnsen et al., 2019), and in the transferrin receptor system, only about 5% of systemically administered doses of NPs reach the CNS and even less reach target cells (Saraiva et al., 2016). Therefore, nasal administration is of increasing interest as an option for NPs to enter the brain and bypass the BBB and also avoid many restrictions of systemic administration (Sousa et al., 2019). However, limited dosing and factors such as nasal congestion and mucus in patients pose significant barriers to the nasal route of drug delivery (Bruinsmann et al., 2019). Choosing the best route of administration for NPs may make the distribution of NPs more ideal, but many current ways of administration will eventually lead to the widespread distribution of NPs. Therefore, overcoming the obstacles of systemic and local drug delivery and increasing drug accumulation at the target site is the focus of future research on the use of targeted NPs in the treatment of gliomas.

Nano-drug delivery systems have been in clinical use since the early 1990s. Doxil^®^, the first FDA-approved nano-drug (Barenholz, 2012), was a milestone in the development of the field of nanomedicine. In the past few decades, a new generation NPs has emerged, and in the current clinical landscape, many NPs have entered clinical trials and have been approved for various indications (Anselmo and Mitragotri, 2019). SGT, a tumor-targeting immunoliposome complex, has been shown to effectively target primary and metastatic tumors in animal models during systemic administration, including targeting brain tumors across the BBB. SGT-53, a nanocomplex containing a normal human wt p53 cDNA, demonstrated excellent tolerability in the first human tests and was followed by a phase 1b combination trial that established the safety and therapeutic potential of SGT-53 when used in combination with docetaxel for the treatment of various solid tumors, including glioblastoma among others (Pirollo et al., 2016). Kim et al. (2018) combined SGT-53 with anti-programmed cell death protein 1 (PD1) antibody and demonstrated that SGT-53 sensitized otherwise refractory tumors to anti-PD1 antibody in three mouse tumor models, including glioblastoma. In later data, SGT-53 was shown to enhance antitumor immunity and sensitize glioblastoma to anti-PD-1 therapies by converting immunologically “cold” tumors into “hot” tumors (Kim et al., 2018). This combination of SGT-53 and anti-PD-1 therapy may benefit more glioma patients from anti-PD-1 immunotherapy. Thus, the combination of NPs with chemotherapy or immunotherapy may bring new prospects for glioma patients, and this combination treatment strategy will be the focus of future research and hot spot for the treatment of glioma.

Nano-drugs have been very extensively studied and have produced good results *in vitro* and in animal models, but the number of therapeutic nano-drugs available to patients is much lower than expected, partly due to the translational gap between animal and human studies (Mitragotri et al., 2017). To fully realize the transformation from laboratory to clinic will be challenging. Understanding the relationship between biology and technology, including the effects of disease pathophysiology on the aggregation, distribution, retention, and efficacy of nanomedicine, and the correlation between the behavior of biopharmaceuticals in animals and humans is an important determinant of the successful transformation of nanomedicine (Hua et al., 2018). The absence of comprehensive studies to understand the correlation between nanomedicine behavior and patient biology in specific clinical applications, as well as disease heterogeneity among patients, may also be a major reason for the failure of promising nano-drug translations in clinical trials (Hare et al., 2017). Therefore, emphasis on the biological basis of the disease and the heterogeneity among patients is the basis for nanomedicines to achieve clinical translation and is the focus of future research.

The vigorous development of nanotechnology has had a great impact on the field of medicine. The application of nano-drug delivery technology has reduced the mortality of cancer. Compared with conventional therapy, such as chemotherapy and radiotherapy, the binding of target molecules on the surface of NPs can enhance their affinity at the tumor site and the ability to cross the BBB, which makes nanomedicine unlimited possibilities for the diagnosis and treatment of gliomas. In this study, we conducted a bibliometric analysis of research on the application of nanotechnology in glioma, which will enable researchers to understand the research trends and hot spots in this field.

### Limitations

1) Data were derived from WOS only, resulting in the omission of publications from other sources. 2) We collected relevant literature from 2012 to June 2022, while the WOS literature is continuously updated. 3) Manual removal of irrelevant publications by reviewers may lead to selection bias.

## Conclusion

We analyzed publications using multiple scientometric tools and revealed bibliometric features of nanotechnology in the glioma field. In addition, a comprehensive analysis of publications in this field was conducted to identify advances in research works and research hotspots in the field. Nanotechnology in glioma treatment still needs to continue to be studied to provide new hope for the therapy of glioma.
