# Supplementary material for: Bibliometric and visualized analysis of the application of nanotechnology in glioma
Source: Front Pharmacol. 2022 Sep 15;13:995512. doi: 10.3389/fphar.2022.995512 (PMC9520472; doi:10.3389/fphar.2022.995512)
Supplement: Supplementary file 1 [file DataSheet2.PDF]

Table S1. Compound annual growth rate of publications 2012– June 2022

Year	No. of publications	Cumulative total	CAGR
2012	83	83	-
2013	114	197	54.06%
2014	139	336	59.38%
2015	137	473	54.51%
2016	195	668	51.75%
2017	212	880	48.22%
2018	201	1081	44.29%
2019	256	1337	41.54%
2020	287	1624	39.16%
2021	297	1921	36.91%
2022	120	2041	33.79%

Table S2. Relative growth rate (RGR) and doubling time (DT) of publications

Year	No. of publications	Cumulative total	W_1	W_2	RGR	DT
2012	83	83		4.4188		
2013	114	197	4.4188	5.2832	0.8644	0.8017
2014	139	336	5.2832	5.8171	0.5339	1.2980
2015	137	473	5.8171	6.1591	0.3420	2.0264
2016	195	668	6.1591	6.5043	0.3452	2.0076
2017	212	880	6.5043	6.7799	0.2756	2.5142
2018	201	1081	6.7799	6.9856	0.2057	3.3687
2019	256	1337	6.9856	7.1982	0.2125	3.2605
2020	287	1624	7.1982	7.3926	0.1945	3.5636
2021	297	1921	7.3926	7.5606	0.1680	4.1261
2022	120	2041	7.5606	7.6212	0.0606	11.4368

Table S3. Top 10 authors and co-cited authors in relevant publications

Rank	Count	Centrality	Authours	Rank	Count	Centrality	Co-cited Authours
1	30	0.01	HUILE GAO	1	412	0.02	STUPP R
2	30	0.02	XINGUO JIANG	2	224	0.16	GAO HL
3	19	0	ZHIQING PANG	3	215	0.02	PARDRIDGE WM
4	17	0	JUN CHEN	4	172	0	OSTROM QT
5	14	0.01	WEIYUE LU	5	154	0.01	LOUIS DN
6	11	0	CHEN JIANG	6	146	0.01	WEN PY
7	11	0	JORDAN J GREEN	7	144	0.27	ABBOTT NJ
8	11	0	YUNGCHIH KUO	8	142	0.06	XIN HL
9	11	0	ZHI YANG	9	138	0.08	LIU Y
10	10	0	SHUANG ZHANG	10	138	0.01	ZHANG Y

Table S4. Top 10 co-cited reference

Rank	Count	Year	Cited References
1	70	2016	The 2016 World Health Organization Classification of Tumors of the Central Nervous System: a summary
2	64	2015	Overcoming the blood-brain tumor barrier for effective glioblastoma treatment
3	57	2016	Nanoparticle-mediated brain drug delivery: Overcoming blood-brain barrier to treat neurodegenerative diseases
4	49	2011	Angiopep-conjugated poly (ethylene glycol)-co-poly(ε-caprolactone) nanoparticles as dual-targeting drug delivery system for brain glioma
5	47	2016	Nanocarriers for the treatment of glioblastoma multiforme: Current state-of-the-art
6	40	2020	The blood-brain barrier and blood-tumour barrier in brain tumours and metastases
7	39	2016	Glioblastoma: Overview of Disease and Treatment
8	39	2012	Anti-glioblastoma efficacy and safety of paclitaxel-loading Angiopep-conjugated dual targeting PEG-PCL nanoparticles
9	38	2016	Progress and perspectives on targeting nanoparticles for brain drug delivery
10	38	2016	Blood-Brain-Barrier-Penetrating Albumin Nanoparticles for Biomimetic Drug Delivery via Albumin-Binding Protein Pathways for Antiglioma Therapy

Table S5. Top 10 keywords for related publications

Rank	Count	Centrality	Year	keywords
1	487	0.01	2012	drug delivery
2	450	0	2012	nanoparticle
3	339	0	2012	in vitro
4	330	0	2012	delivery
5	285	0.01	2012	cancer
6	266	0	2012	blood brain barrier
7	246	0	2012	therapy
8	221	0	2012	cell
9	203	0.01	2012	glioblastoma
10	184	0	2012	glioma
