# Supplementary material for: Bibliometric and visualized analysis of the application of nanotechnology in glioma
Source: Front Pharmacol. 2022 Sep 15;13:995512. doi: 10.3389/fphar.2022.995512 (PMC9520472; doi:10.3389/fphar.2022.995512)
Supplement: Supplementary file 2 [file DataSheet1.PDF]

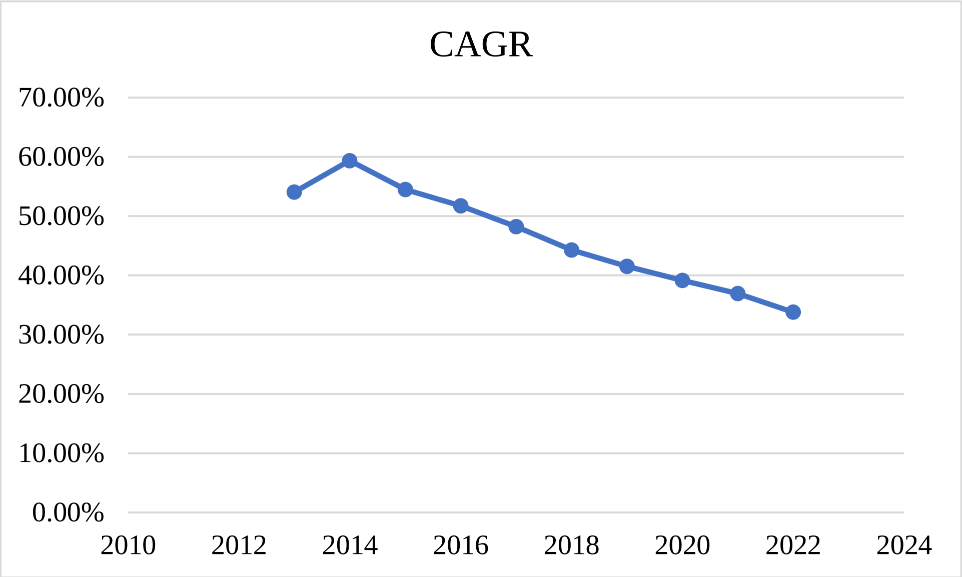

Fig.S1. Compound annual growth rate (CAGR) of publications.

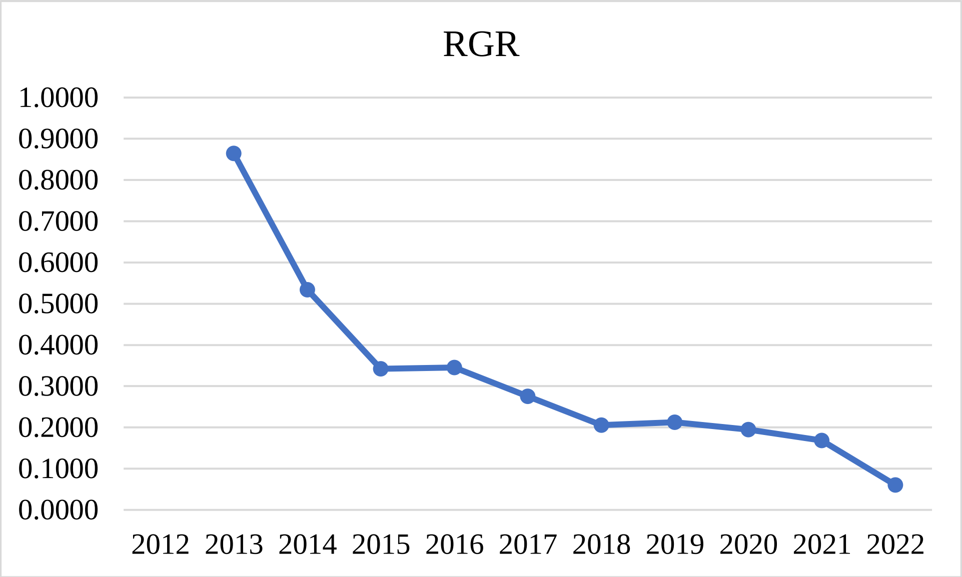

Fig. S2. Relative growth rate (RGR) of publications.

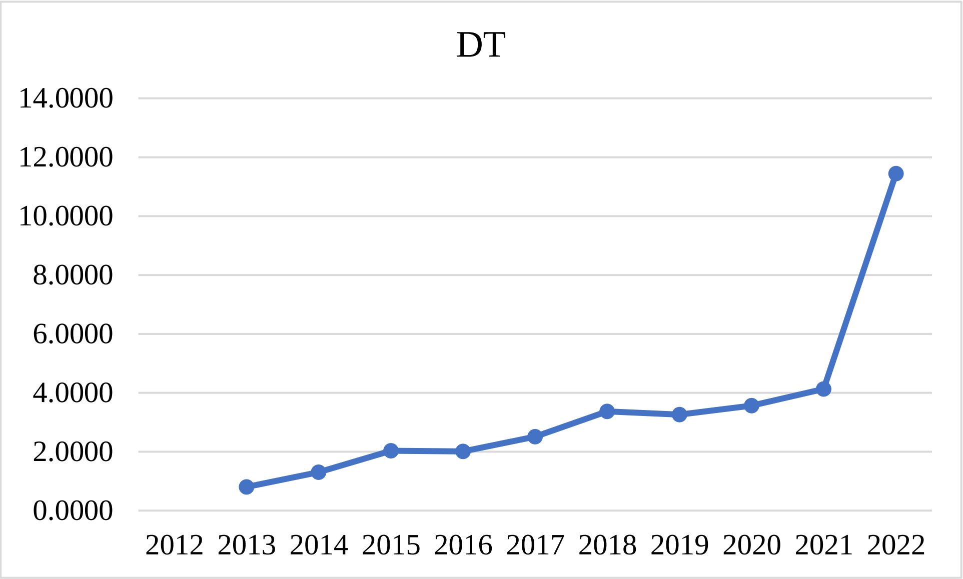

Fig. S3. Doubling time (DT) of publications.

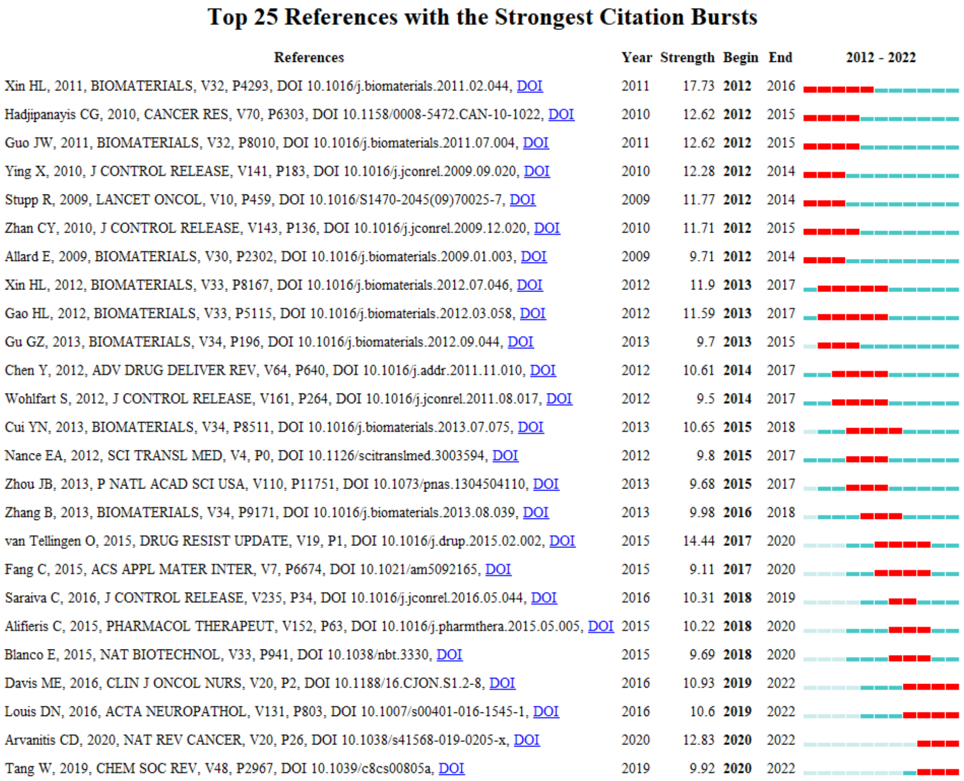

Top 25 References with the Strongest Citation Bursts

References	Year	Strength	Begin	End	2012 - 2022
Xin HL, 2011, BIOMATERIALS, V32, P4293, DOI 10.1016/j.biomaterials.2011.02.044, DOI	2011	17.73	**2012**	2016	
Hadjipanayis CG, 2010, CANCER RES, V70, P6303, DOI 10.1158/0008-5472.CAN-10-1022, DOI	2010	12.62	**2012**	2015	
Guo JW, 2011, BIOMATERIALS, V32, P8010, DOI 10.1016/j.biomaterials.2011.07.004, DOI	2011	12.62	**2012**	2015	
Ying X, 2010, J CONTROL RELEASE, V141, P183, DOI 10.1016/j.jconrel.2009.09.020, DOI	2010	12.28	**2012**	2014	
Stupp R, 2009, LANCET ONCOL, V10, P459, DOI 10.1016/S1470-2045(09)70025-7, DOI	2009	11.77	**2012**	2014	
Zhan CY, 2010, J CONTROL RELEASE, V143, P136, DOI 10.1016/j.jconrel.2009.12.020, DOI	2010	11.71	**2012**	2015	
Allard E, 2009, BIOMATERIALS, V30, P2302, DOI 10.1016/j.biomaterials.2009.01.003, DOI	2009	9.71	**2012**	2014	
Xin HL, 2012, BIOMATERIALS, V33, P8167, DOI 10.1016/j.biomaterials.2012.07.046, DOI	2012	11.9	**2013**	2017	
Gao HL, 2012, BIOMATERIALS, V33, P5115, DOI 10.1016/j.biomaterials.2012.03.058, DOI	2012	11.59	**2013**	2017	
Gu GZ, 2013, BIOMATERIALS, V34, P196, DOI 10.1016/j.biomaterials.2012.09.044, DOI	2013	9.7	**2013**	2015	
Chen Y, 2012, ADV DRUG DELIVER REV, V64, P640, DOI 10.1016/j.addr.2011.11.010, DOI	2012	10.61	**2014**	2017	
Wohlfart S, 2012, J CONTROL RELEASE, V161, P264, DOI 10.1016/j.jconrel.2011.08.017, DOI	2012	9.5	**2014**	2017	
Cui YN, 2013, BIOMATERIALS, V34, P8511, DOI 10.1016/j.biomaterials.2013.07.075, DOI	2013	10.65	**2015**	2018	
Nance EA, 2012, SCI TRANSL MED, V4, P0, DOI 10.1126/scitranslmed.3003594, DOI	2012	9.8	**2015**	2017	
Zhou JB, 2013, P NATL ACAD SCI USA, V110, P11751, DOI 10.1073/pnas.1304504110, DOI	2013	9.68	**2015**	2017	
Zhang B, 2013, BIOMATERIALS, V34, P9171, DOI 10.1016/j.biomaterials.2013.08.039, DOI	2013	9.98	**2016**	2018	
van Tellingen O, 2015, DRUG RESIST UPDATE, V19, P1, DOI 10.1016/j.drup.2015.02.002, DOI	2015	14.44	**2017**	2020	
Fang C, 2015, ACS APPL MATER INTER, V7, P6674, DOI 10.1021/am5092165, DOI	2015	9.11	**2017**	2020	
Saraiva C, 2016, J CONTROL RELEASE, V235, P34, DOI 10.1016/j.jconrel.2016.05.044, DOI	2016	10.31	**2018**	2019	
Alifieris C, 2015, PHARMACOL THERAPEUT, V152, P63, DOI 10.1016/j.pharmthera.2015.05.005, DOI	2015	10.22	**2018**	2020	
Blanco E, 2015, NAT BIOTECHNOL, V33, P941, DOI 10.1038/nbt.3330, DOI	2015	9.69	**2018**	2020	
Davis ME, 2016, CLIN J ONCOL NURS, V20, P2, DOI 10.1188/16.CJON.S1.2-8, DOI	2016	10.93	**2019**	2022	
Louis DN, 2016, ACTA NEUROPATHOL, V131, P803, DOI 10.1007/s00401-016-1545-1, DOI	2016	10.6	**2019**	2022	
Arvanitis CD, 2020, NAT REV CANCER, V20, P26, DOI 10.1038/s41568-019-0205-x, DOI	2020	12.83	**2020**	2022	
Tang W, 2019, CHEM SOC REV, V48, P2967, DOI 10.1039/c8cs00805a, DOI	2019	9.92	**2020**	2022	

Fig. S4. CiteSpace visualization map of top 25 references with the strongest citation bursts.

Top 25 Keywords with the Strongest Citation Bursts

Keywords	Year	Strength	Begin	End
brain tumor	2012	8.72	**2012**	2013
in vivo	2012	7.68	**2012**	2013
malignant glioma	2012	6.49	**2012**	2013
multidrug resistance	2012	6.06	**2012**	2016
gene delivery	2012	5.92	**2012**	2016
biodistribution	2012	5.69	**2012**	2015
endothelial cell	2012	3.96	**2012**	2014
receptor	2012	3.66	**2012**	2013
tumor cell	2012	4.36	**2013**	2016
monoclonal antibody	2012	3.56	**2013**	2016
therapeutics	2012	4.47	**2014**	2015
antitumor activity	2012	4.25	**2014**	2016
liposomal doxorubicin	2012	3.91	**2014**	2015
extracellular matrix	2012	3.45	**2014**	2017
microvascular endothelial cell	2012	4.18	**2015**	2016
formulation	2012	4.16	**2016**	2017
cancer therapy	2012	3.74	**2016**	2017
solid tumor	2012	3.52	**2016**	2018
angiopep 2	2012	3.31	**2017**	2018
photodynamic therapy	2012	4.96	**2018**	2022
autophagy	2012	3.57	**2018**	2019
resection	2012	5.14	**2019**	2020
functionalization	2012	3.81	**2019**	2020
optimization	2012	3.7	**2019**	2022
macrophage	2012	3.53	**2020**	2022

Fig.S5. Top 25 keywords with the strongest citation bursts.

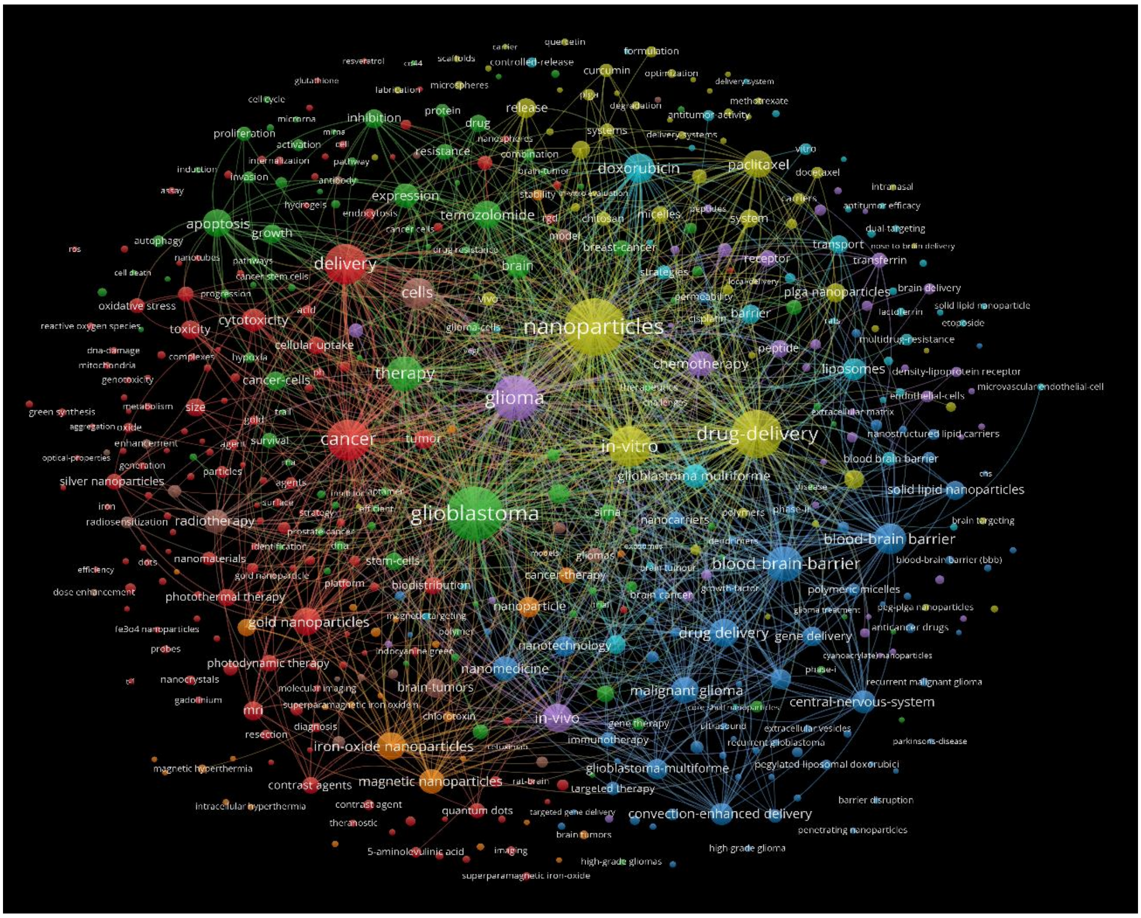

Fig. S6. VOSviewer visualization for keywords clustering.
